# Concurrent AESOP and POEM syndrome presenting as an indurated plaque: AESOP’s paraneoplastic POEM

**DOI:** 10.1016/j.jdcr.2025.06.029

**Published:** 2025-06-28

**Authors:** Aaron Cheng, Harrison Shawa, Brian Hinds, David G. Cotter

**Affiliations:** a Long School of Medicine, University of Texas Health San Antonio, San Antonio, Texas; b Division of Dermatology, Washington University in St. Louis, St. Louis, Missouri; c Department of Dermatology, University of California, San Diego, San Diego, California; d Las Vegas Dermatology, Las Vegas, Nevada; e Department of Internal Medicine, University of Nevada Las Vegas School of Medicine, Las Vegas, Nevada

**Keywords:** AESOP syndrome, plasmacytoma, POEMS syndrome

## Introduction

Adenopathy and extensive skin patch overlying a plasmacytoma (AESOP) is a rare paraneoplastic syndrome primarily associated with osseous plasma cell neoplasms.^1^ AESOP syndrome presents with an indurated, erythematous patch, but it is often linked to symptoms of polyneuropathy, organomegaly, endocrinopathy, monoclonal paraproteinemia, and skin changes, referred to as POEMS syndrome.^1^ Early recognition can be lifesaving; however, the obscure and seemingly unrelated concurrent presentations of these syndromes can make a diagnosis of AESOP-POEMS challenging. Herein, we present a novel case of AESOP-POEMS syndrome, highlighting its distinctive clinical manifestations and underlying pathophysiological mechanisms.

## Case report

**Fig 1:**
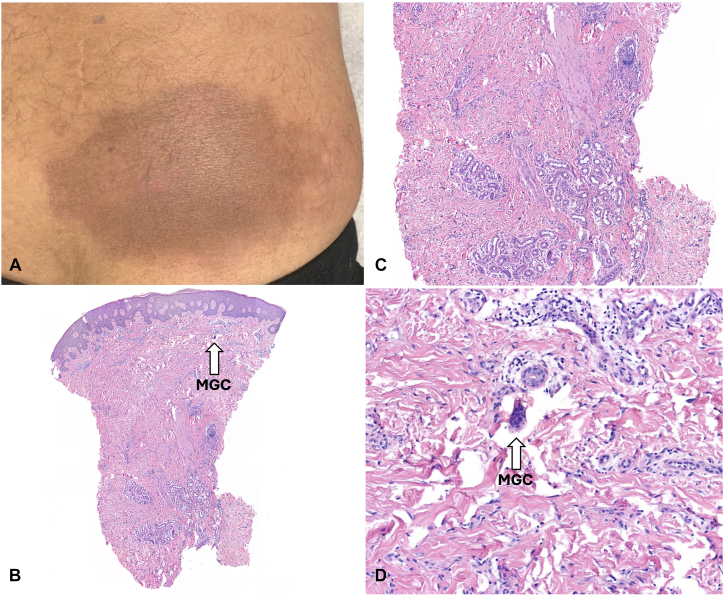
**A,** Clinical image of an indurated, warm, and erythematous *brown-pink* plaque overlying the right iliac crest. **B-D,** Hematoxylin-eosin-stained specimen from skin lesion showing fibrovascular proliferation, floret-like multinucleate giant cells (MGC), and slightly increased dermal mucin.

A 53-year-old man presented with a 6-month history of an indurated, warm, and erythematous brown-pink plaque overlying his right iliac spine (Fig 1, *A*). Previous misdiagnoses of tinea corporis and herpes zoster led to treatment with antifungal and oral antiviral regimens without benefit. Approximately 2-months after the skin change appeared, the patient reported a series of unexplained symptoms, including weakness, 90-pound weight loss, hypogonadism, thyroid dysfunction, bilateral lower extremity neuropathy, weakness precluding ambulation, and motor dysfunction.

Upon presentation to our clinic, the patient was cachectic and wheelchair confined. Previous laboratory results showed elevated platelets to 522,000 cells/μL (reference range: 150,000-450,000 cells/μL), thyroid-stimulating hormone, alkaline phosphatase to 132 IU/L (reference range: 40-116 IU/L), smooth muscle actin antibodies to 24 U (reference range: <20 U), and vascular endothelial growth factor (VEGF) to 9457 pg/mL (reference range: 31-86 pg/mL). Additional workup revealed low free testosterone, proteinuria, and abnormal immunofixation with M-spike on serum protein electrophoresis. More specifically, immunofixation showed an IgG monoclonal protein with lambda light chain specificity.

Histopathologic examination revealed a fibrovascular proliferation, floret-like multinucleate cells, and slightly increased dermal mucin (Fig 1, *B-D*). A CT scan revealed expansile, mixed lytic and sclerotic lesions in the posterior aspect of the right iliac crest. Following imaging, a bone marrow biopsy revealed a plasmacytoma, thus confirming the diagnosis of AESOP. Combined with the patient’s history of polyneuropathy, hypogonadism, thyroid dysfunction, M-spike, VEGF elevation, and skin changes, a concurrent diagnosis of POEMS syndrome was established.

The patient underwent treatment for the plasmacytoma, receiving a combination of radiation, bortezomib, lenalidomide, and physical therapy. At 3-month follow-up, the plaque overlying the plasmacytoma reduced in size, his lower extremity neuropathy and motor dysfunction had improved, and he could ambulate independently.

## Discussion

We present a complex clinical presentation of AESOP syndrome with concurrent POEMS syndrome. AESOP-POEMS syndromes are paraneoplastic phenomena associated with plasma cell neoplasms such as multiple myeloma and lymphoplasmacytic lymphoma.^2^ There are 2 clinical variants of AESOP syndrome, which share manifestations similar to other diseases, making diagnosis challenging.^3^ The classic variant typically presents with a red-to-brown macule that is shiny, translucent, smooth, slowly expanding, and first appears over a region where the skin is directly superficial to bone^1^ (ie, sternum, skull, flank regions). The morphea-like variant presents with slightly infiltrated, pinkish brown lesions, with 70% of the patients experiencing concurrent sensory-motor neuropathy.^3^ Histologically, the majority of cases show fibrovascular proliferation and relatively dramatic mucin deposition^4^ (Fig 1, *B*). Another distinctive feature of AESOP syndrome, especially the morphea-like variant, is its association with monoclonal gammopathy, which is also a hallmark of POEMS syndrome.^5^

The combination of clinical features seen in POEMS syndrome is complex. Patients with POEMS syndrome must fulfill both mandatory and minor criteria, with at least 1 of 3 major criteria and 1 of 6 minor criteria.^6^ Mandatory criteria include polyneuropathy, which is typically demyelinating, a monoclonal plasma cell-proliferative disorder, like Castleman disease or sclerotic plasmocytic bone lesions, and VEGF elevation.^6^^,^^7^ Minor criteria include organomegaly (splenomegaly, lymphadenopathy, or hepatomegaly), extravascular volume overload (edema, pleural effusion, or ascites), endocrinopathy (adrenal, thyroid, pituitary, gonadal, parathyroid, pancreatic), skin changes (hyperpigmentation, hypertrichosis, glomeruloid hemangiomata, plethora, acrocyanosis, flushing, leukonychia), papilledema, and thrombocytosis and polycythemia.^6^^,^^7^ Other signs and symptoms can include clubbing, weight loss, restrictive lung disease, and thrombotic diatheses.^6^ Our patient met all criteria for POEMS syndrome.

The exact mechanisms underlying the pathogenesis of POEMS syndrome are still unclear. Due to its high association with plasma cell malignancies, pro-inflammatory cytokines such as VEGF, IL-1 β, and interleukin-6 secreted by the neoplastic cells are proposed to be major mediators of the disease.^8^ Previous reports have suggested a correlation between high VEGF levels and the skin changes seen in POEMS syndrome.^9^ The paraneoplastic effects of VEGF, causing endothelial cell proliferation and angiogenesis, may explain the skin changes seen in our case. However, previous cases suggest that the prognostic value of VEGF is insufficient, as the anti-VEGF monoclonal antibody treatment, bevacizumab, resulted in almost no clinical response.^10^ Current management strategies prioritize treating the underlying plasmacytoma with radiation therapy and additional systemic therapies such as bortezomib and lenalidomide.^8^

In conclusion, we present a rare case of concurrent AESOP and POEMS syndrome that had been previously misdiagnosed. This case highlights the diagnostic challenges in identifying AESOP with overlapping paraneoplastic syndromes, emphasizing the importance of comprehensive clinical evaluation and awareness of these rare conditions. AESOP syndrome can be used as an early warning sign for the more life-threatening POEMS syndrome, given their close association. Therefore, keeping a high clinical suspicion for AESOP and POEMS is crucial when presented with an enlarging patch or plaque overlying a bony area, especially in patients with accompanying systemic symptoms.

## Conflicts of interest

None disclosed.
